# The association between exposure to famine in early life and risks of diabetic complications in adult patients with type two diabetes

**DOI:** 10.7189/jogh.14.04167

**Published:** 2024-09-20

**Authors:** Chu Lin, Xiaoling Cai, Zonglin Li, Fang Lv, Wenjia Yang, Linong Ji

**Affiliations:** Department of Endocrinology and Metabolism, Peking University People’s Hospital, Beijing, China

## Abstract

**Background:**

In this study, we aimed to assess the associations between early exposure to famine and the risks of diabetic complications in adult patients with type two diabetes.

**Methods:**

The participants in this study were selected from China National HbA1c Surveillance System (2009–13) and further stratified according to the birth year. The participants born between 1956–59, 1959–61, and 1962–64 were classified as foetal exposed group with 70 852, infant/toddler exposed group with 93 616, and unexposed group with 72 723 participants. The association between exposure to famine in early life and risks of diabetic complications were analysed by logistic regression. We assessed the attributing effects of the interaction between exposure to famine in early life and modifiable risk factors by the multiplicative and additive interactive models.

**Results:**

After adjustments for sex, famine severity, economic status in adulthood, body mass index, blood pressure, low-density lipoprotein cholesterol, glycated haemoglobin, diabetes duration, and the use of antidiabetic agents, the increased risks of coronary heart disease (odds ratio (OR) = 1.31; 95% CI (confidence interval) = 1.26, 1.36), cerebrovascular disease (OR = 1.32; 95% CI = 1.24, 1.41), and diabetic retinopathy (OR = 1.06; 95% CI = 1.02, 1.10) were observed in patients with early-life exposure to famine. The reduced risk of diabetic kidney disease (OR = 0.94; 95% CI = 0.90, 0.99) was observed in patients with early-life exposure to famine compared with those without famine exposure. The interaction analyses indicated that obesity might exacerbate the increased risk of coronary heart disease (OR = 1.26; 95% CI = 1.22, 1.30), cerebrovascular disease (OR = 1.26; 95% CI = 1.21, 1.32), and diabetic retinopathy associated with early-life exposure to famine (OR = 1.09; 95% CI = 1.06, 1.12) in patients with type two diabetes. Moreover, high economic status in adulthood might also exacerbate the increased risk of coronary heart disease (OR = 1.35; 95% CI = 1.30, 1.40) and cerebrovascular disease (OR = 1.33; 95% CI = 1.23, 1.43) associated with early-life exposure to famine in patients with type two diabetes.

**Conclusions:**

Early-life exposure to famine in patients with type two diabetes might be associated with increased risks of coronary heart disease, cerebrovascular disease, and diabetic retinopathy but a reduced risk of diabetic kidney disease in adulthood. Obesity and high economic status might further exacerbate the risk of diabetic complications associated with early-life exposure to famine. Improving early-life nutritional status may promote better risk prevention and management of diabetic complications in patients with type two diabetes.

Type two diabetes (T2D) is a common non-communicable disease. The prevalence of T2D has been increasing annually in China due to economic development, urbanisation, and the ageing population. The pandemic of overweight and obesity further contributes to this trend [1]. Although numerous epidemiological studies have shown that factors such as overweight or obesity, high-salt or high-fat diets, sedentary lifestyles, and lack of exercise are associated with an increased risk of T2D, these factors alone cannot fully explain the dramatical increase in the prevalence of T2D in China over the past two decades [2,3].

Tracing back in history, researchers found clues in the Chinese three-year natural disasters caused by famine from 1959 to 1961. According to the Developmental Origins of Health and Disease (DOHaD) theory, individuals born during the Great Famine underwent structural and metabolic adjustments in early life to adapt to environmental pressure [4]. These adjustments enabled them to cope with the environmental stressors and increased the risk of developing various non-communicable diseases later in life. Previous studies suggested that exposure to famine during early life may affect the metabolic regulatory mechanisms through epigenetic effects, consequently altering the trajectory of T2D prevalence in China [5–7].

However, the exploration of the association between early-life famine exposure and metabolic diseases has primarily been conducted in the general population. Although previous studies suggest that early-life exposure to famine is associated with increased risk of T2D, hypertension, obesity and other metabolic diseases, the relationship between exposure to famine during early life and the risk of diabetic complications in patients with T2D is less characterised and remains unclear [8,9]. Therefore, we designed and conducted this study aims to explore the impact of early-life famine exposure on the risk of diabetic complications (macrovascular complications: coronary heart disease, cerebrovascular disease; microvascular complications: diabetic retinopathy, diabetic nephropathy, diabetic peripheral neuropathy, diabetic foot) in Chinese population with T2D.

## METHODS

### Study population

The China National HbA1c Surveillance System (CNHSS) project is a nationwide cross-sectional study initiated by the Chinese Diabetes Society of the Chinese Medical Association [10,11]. This study was conducted from 2009 to 2013 and selected representative key hospitals (including tertiary, secondary, and community hospitals or health service centres) from various provinces, municipalities, and autonomous regions nationwide on designated visit days. The recruitment period for this project was three months each year, starting in March and ending on 30 June. Each key hospital recruited up to 400 patients or until the recruitment deadline. The number of cities participating in the CNHSS project from 2009 to 2013 were 89, 97, 129, 104, and 84, respectively. The number of participating hospitals were 417, 347, 606, 624, and 586, respectively.

The inclusion criteria for the CNHSS study were as follows: 1) outpatients with T2D aged ≥18 years, 2) patients receiving treatment with oral hypoglycaemic drugs or a combination of oral drugs and insulin, or a combination of oral drugs and glucagon-like peptide-1 receptor agonists (GLP-1RA) (individuals solely using insulin or GLP-1RA have also been included since 2012), 3) patients with at least one complete medical record from an outpatient or hospital visit for T2D, and 4) local residents, who have lived in the local area for at least six months.

The exclusion criteria for the CNHSS study were as follows: 1) type one diabetes, 2) diabetes secondary to other diseases, 3) patients sorely adopting lifestyle interventions, 4) patients sorely treated with traditional Chinese medicine, 5) patients currently receiving inpatient treatment, 6) women during the pregnancy or lactation period, and 7) patients with impaired consciousness, inability to communicate normally, or unable to cooperate with the investigation due to other reasons.

Ethics approval for CNHSS was obtained from the ethics committee for clinical research of the People’s Liberation Army General Hospital. Informed consent was obtained by signing the official consent paperwork before participant data was collected. Ethics approval for this famine study was obtained from the ethics committee of Peking University People’s Hospital.

### Famine exposure grouping

**Figure 1:**
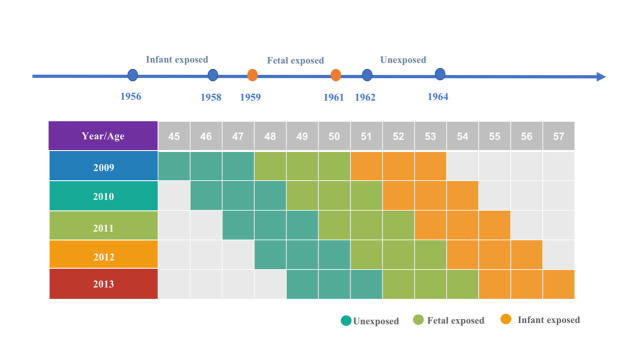
The stratifications of famine exposure.

Previous epidemiological studies on Chinese famines have utilised varying methods for grouping famine exposure. Still, most studies have considered three years of natural disasters from 1959 to 1961 as the time of famine or included the years 1959–61 as the famine period [12]. Since specific birth dates of participants were not collected in the CNHSS study, we used the participants’ age as a proxy variable. Participants born between 1959–61 were defined as the foetal period exposed group, those born between 1956–58 were defined as the infancy exposed group, and those born between 1962–64 were defined as the non-exposed group. The combined group of foetal-exposed and infancy-exposed participants was defined as the early-life exposed group. The age distribution for each group is shown in **Figure 1**.

### Severity of famine exposure

The excess death rate (EDR) for each province/municipality/autonomous region during China’s three years of natural disasters was used as a proxy variable for famine severity. The EDR data used in this study was sourced from the special report ‘Famine and Overweight in China’ [13]. The report defined EDR as the relative change in the highest mortality rate between 1959–62 compared to the average mortality rate from 1956–5858 in China [13]. We defined provinces/municipalities/autonomous regions with EDR≥100% as areas with relatively severe famine and those with EDR<100% as areas with relatively mild famine (Table S1 in the **Online Supplementary Document****).**

### Economic status in adulthood

We introduced the variable of economic status in adulthood, measured by the annual per capita disposable income of residents in each province/municipality/autonomous region. We compared it with each year’s national average per capita disposable income. Areas with per capita disposable income higher than the national average were defined as having relatively high economic status, while areas with per capita disposable income lower than the national average were defined as having low economic status. The resident income data used in this study were derived from the China Economic and Financial Research Database. The urban and rural population data were obtained from the statistical yearbooks of China published by the National Bureau of Statistics for the years 2009–13 (Table S2 in the **Online Supplementary Document**).

### Population migration rate

Given that the CNHSS study defined permanent residents as those who have lived in the local area for six months or more, it cannot fully distinguish the birthplace and place of residence of the subjects. To reduce the impact of population migration on this study, this study included the variable of population migration rate to adjust the subsequent analysis process. Based on the data of the sixth national population census in 2010 [14], the population migration rate was defined as the percentage of the people whose birthplace and place of residence were not in the same area, accounting for the total population of the area and taking 10% as the cut-off point. The area with a population migration rate ≥10% was classified as the area with a high population migration rate. The area with a population migration rate <10% was classified as the area with a low population migration rate (Table S3 in the **Online Supplementary Document**).

### Diabetic complications in the study

The diabetic complications involved in this study refer to newly occurring conditions after the diagnosis of T2D. These complications include macrovascular complications, including coronary heart disease and cerebrovascular disease, microvascular complications, including diabetic retinopathy and diabetic kidney disease, diabetic peripheral neuropathy, and diabetic foot [10,11]. The definitions of the aforementioned clinical outcomes or events are summarised in Table S4 in the **Online Supplementary Document**.

### Statistical analysis methods

Continuous variables were expressed as means (x̄) and standard deviations (SD). Independent sample *t* tests were used for comparisons between two groups, while one-way analysis of variance was used for comparisons among multiple groups. For data with homogeneous variances, the least significant difference analysis method was used, and for data with heterogeneous variances, Dunnett’s T3 method was adopted. Discontinuous variables were expressed as percentages. We used χ^2^ tests for comparisons between groups, with Bonferroni correction applied for multiple comparisons. We used logistic regression analysis to evaluate the influence of famine exposure on the risk of diabetic complications. Further, we conducted an interaction analysis to evaluate the interactive effects of famine exposure on additional modifiable risk factors [15]. Relative excess risk due to interaction (RERI), attributable proportion due to interaction (AP), and synergy index (S) were calculated using the formulations developed by Andersson et al. [16]. If there is no additive interaction between the two factors, the confidence intervals of RERI and AP includes ‘zero’, and the confidence interval of S will includes ‘one’.

Data analysis for this study was performed using SPSS, version 23.0 (IBM, Armonk, New York, USA). *P*-value <0.05 was considered statistically significant, and all tests were two-tailed.

## RESULTS

### Clinical characteristics of the study population

A total of 237 191 individuals were included in this analysis. Among them, 72 723 individuals were in the unexposed group, 70 852 were in the foetal-exposure group, 93 616 were in the infant-exposure group, and the combined early-life exposure group consisted of 164 468 individuals. The age distribution in each exposure group was as follows: unexposed group (x̄ = 48.36, SD = 1.51), foetal-exposure group (x̄ = 51.35, SD = 1.57), infant-exposure group (x̄ = 54.45, SD = 1.56). The average age in the combined early-life exposure group was 53.11 years (SD = 2.20), with a difference of approximately five years compared to the unexposed group. The male-to-female ratio was approximately 1:1 in all famine exposure groups (Table S5 in the **Online Supplementary Document**).

### The association between early-life famine exposure and risks of diabetic complications

**Figure 2:**
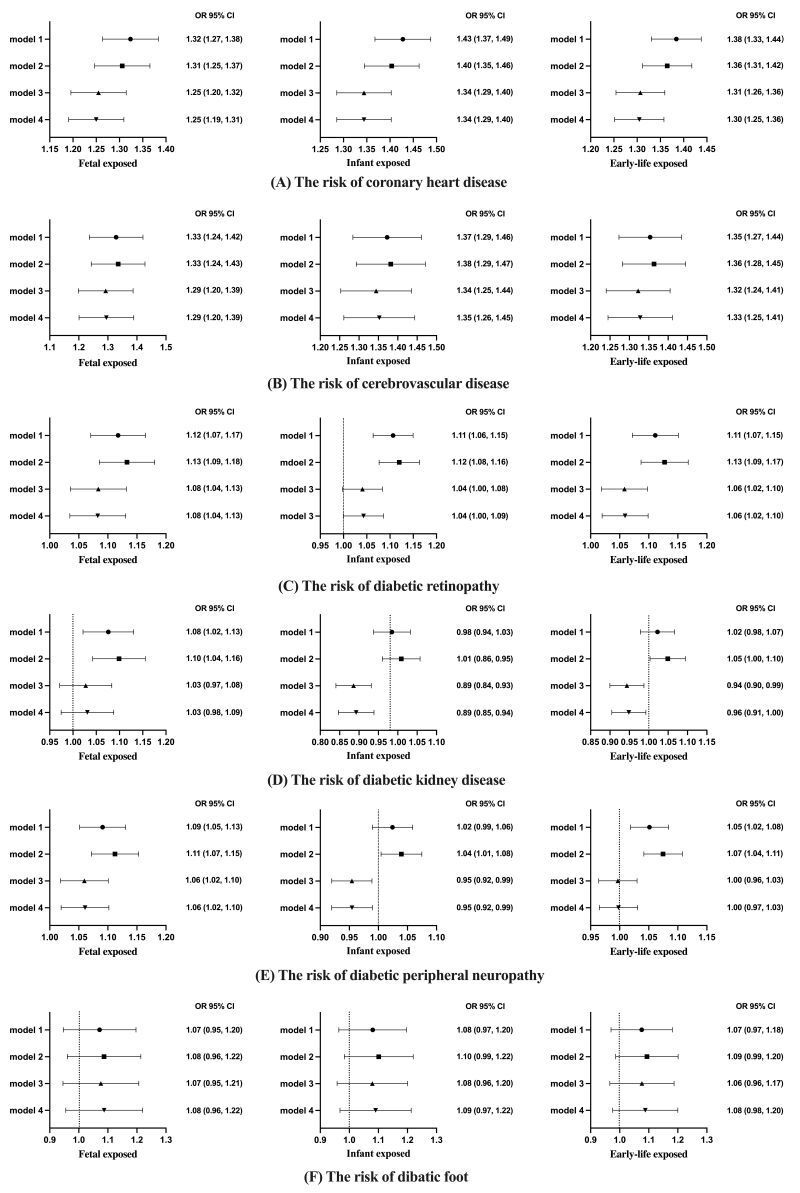
The association between early-life famine exposure and diabetic complications in adulthood. **Panel A.** The risk of coronary heart disease. **Panel B.** The risk of cerebrovascular disease. **Panel C**. The risk of diabetic retinopathy. **Panel D.** The risk of diabetic kidney disease. **Panel E.** The risk of diabetic peripheral neuropathy. **Panel F.** The risk of diabetic foot. Model one – crude evaluation without adjustments. Model two – adjusted for sex, the severity of famine exposure, and economic status in adulthood. Model three – adjusted for sex, body mass index, systolic and diastolic blood pressure, low-density lipoprotein cholesterol, HbA1c, diabetes duration, the severity of famine exposure, economic status in adulthood, the use of metformin, and the use of glucagon-like peptide one receptor agonist. Model four – adjusted for sex, body mass index, targeted rate for blood pressure control, targeted rate for low-density lipoprotein cholesterol control, targeted rate for glycaemic control, diabetes duration, the severity of famine exposure, economic status in adulthood, the use of metformin, and the use of glucagon-like peptide one receptor agonist.

As for coronary heart disease, after the multiple adjustments, compared to the unexposed group, the risks of coronary heart disease were increased in both the foetal-exposure group (odds ratio (OR) = 1.25; 95% confidence interval (CI) = 1.20, 1.32) and infant-exposure group (OR = 1.34; 95% CI = 1.29, 1.40). The early-life famine exposure group had an approximately 30% increased risk of coronary heart disease (OR = 1.31; 95% CI = 1.26, 1.36) compared to those without famine exposure (**Figure 2**, Panel A). Similarly, after adjusting for multiple influencing factors, compared to the unexposed group, the risks of cerebrovascular disease were also increased in both the foetal-exposure group (OR = 1.29; 95% CI = 1.20, 1.39) and the infant-exposure group (OR = 1.34; 95% CI = 1.25, 1.44). The early-life exposure group had an approximately 32% increased risk of cerebrovascular disease (OR = 1.32; 95% CI = 1.24, 1.41) compared to patients without famine exposure (**Figure 2**, Panel B).

Concerning diabetic retinopathy, compared to the unexposed group, a slight increase in the risk of diabetic retinopathy was observed in the foetal-exposure group (OR = 1.08; 95% CI = 1.04, 1.13) and the early-life exposure group (OR = 1.05; 95% CI = 1.02, 1.10), after adjusting for confounding factors (**Figure 2**, Panel C). However, after adjusting for relevant confounding factors, individuals with T2D who experienced famine exposure during infancy had a significantly lower risk of developing diabetic kidney disease in adulthood compared to the unexposed group (OR = 0.89; 95% CI = 0.84, 0.93) (**Figure 2**, Panel D). The analysis of the early-life exposure group also observed similar risk reduction (OR = 0.94; 95% CI = 0.90, 0.99) (**Figure 2**, Panel D).

Interestingly, compared to the unexposed group, the risk of diabetic peripheral neuropathy was increased in the foetal-exposure group (OR = 1.07; 95% CI = 1.02, 1.10) but was reduced in the infant-exposure group (OR = 0.95; 95% CI = 0.92, 0.99) (**Figure 2**, Panel E). Early-life famine exposure did not significantly affect the risk of diabetic foot in individuals with T2D in adulthood (**Figure 2**, Panel F).

### The association between the severity of famine exposure and risks of diabetic complications

**Figure 3:**
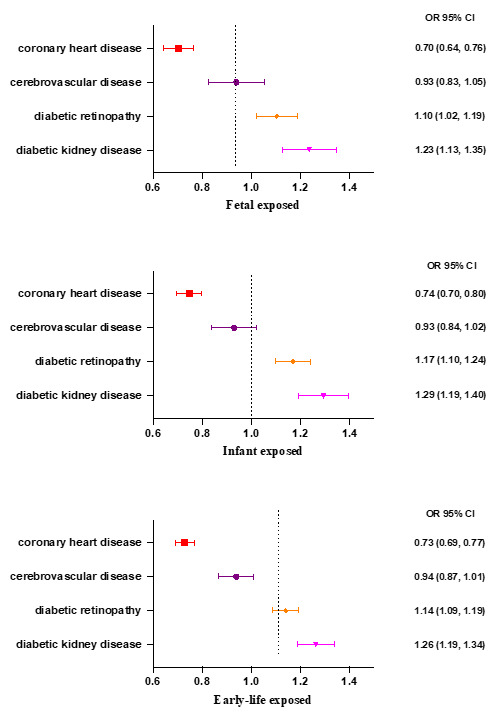
The association between the severity of famine exposure and risks of diabetic complications in adulthood. The model was adjusted for sex, body mass index, systolic and diastolic blood pressure, low-density lipoprotein cholesterol, HbA1c, diabetes duration, economic status in adulthood, the use of metformin, and the use of a glucagon-like peptide one receptor agonist.

In the foetal-exposure group, compared to those in the areas with relatively mild famine severity, individuals born in areas with more severe famine experienced an increased risk of diabetic retinopathy (OR = 1.10; 95% CI = 1.02, 1.19) and diabetic kidney disease (OR = 1.23; 95% CI = 1.13, 1.35) in adulthood, but a decreased risk of coronary heart disease (OR = 0.70; 95% CI = 0.64, 0.76) (**Figure 3**). A similar situation was also observed in the infant-exposure group. The more severe famine exposure seemed to exacerbate the risk of diabetic retinopathy (OR = 1.17; 95% CI = 1.10, 1.24) and diabetic kidney disease (OR = 1.29; 95% CI = 1.19, 1.40) in adulthood but was associated with a decreased risk of coronary heart disease (OR = 0.74; 95% CI = 0.70, 0.80). Consistent results were also observed in the analysis for the early-life famine exposure group (**Figure 3**).

### Sensitivity analyses based on sex, body mass index, disease duration, economic status in adulthood, migration rate and recruiting year

We also performed multiple sensitivity analyses according to sex, obesity (body mass index <28 kg/m^2^ or ≥28 kg/m^2^), duration of diabetes (≥10 years or <10 years), economic status in adulthood (higher/equal to or lower than the national per capita disposable income), and migration rate (≥10% or <10%). It seemed that the increased risks of coronary heart disease, diabetic retinopathy, and diabetic peripheral neuropathy were more profound in females when compared to male patients who underwent early-life famine exposure. Moreover, greater risk increases in diabetic retinopathy and diabetic peripheral neuropathy were observed in patients with a long duration of diabetes vs those with a duration of diabetes below 10 years (Figure S1 in the **Online Supplementary Document**). We also performed the sensitivity analyses according to the recruiting years, and the results were generally consistent with the overall analysis (Figure S2 in the **Online Supplementary Document**).

### Interaction analyses for early-life famine exposure and modifiable risk factors

The additive interaction model suggested an additive interaction between early-life famine exposure and obesity, with an excess risk RERI = 0.19; 95% CI = 0.02, 0.36). It was shown that 9% of the increased risk of coronary heart disease was attributable to the interaction between early-life famine exposure and obesity (AP = 0.09; 95% CI = 0.01, 0.18). When early-life famine exposure and obesity were present together, the risk of coronary heart disease was 1.23 times the sum of the risks when these two factors were considered individually (S = 1.23; 95% CI = 1.01, 1.50) (**Table 1**). Similarly, high socioeconomic status during adulthood might exacerbate the increased risk of coronary heart disease associated with early-life exposure to famine. The additive interaction model suggested an additive interaction between early-life famine exposure and high socioeconomic status during adulthood (RERI = 0.12, 95% CI = 0.02, 0.21; AP = 0.07, 95% CI = 0.01, 0.13; S = 1.23, 95% CI = 1.01, 1.51). The multiplicative interaction model suggested a multiplicative interaction between early-life famine exposure and obesity (OR = 1.26; 95% CI = 1.21, 1.32). Similarly, high socioeconomic status during adulthood might further increase the risk of cerebrovascular disease associated with early-life exposure to famine. However, the interaction analysis suggests the presence of only a multiplicative interaction (OR = 1.33; 95% CI = 1.23, 1.43) and no additive interaction between these two factors. As for diabetic retinopathy, the interaction analysis suggests a multiplicative interaction between early-life famine exposure and obesity on diabetic retinopathy (OR = 1.09; 95% CI = 1.06, 1.12), but no additive interaction was observed (**Table 1**).

**Table 1:** Interaction analyses for early-life famine exposure and modifiable risk factors

Interaction analysis			Obesity, OR (95% CI)*		High economic status, OR (95% CI)†	
Coronary heart disease						
*Single effect for famine exposure*			1.31 (1.26, 1.37)		1.29 (1.20, 1.38)	
*Single effect for risk factor*			1.50 (1.37, 1.64)		1.22 (1.13, 1.31)	
*Combined effect*			2.00 (1.88, 2.13)		1.63 (1.52, 1.73)	
*Multiplicative interaction*			1.26 (1.22, 1.30)		1.35 (1.30, 1.40)	
Additive interaction						
*RERI*			0.19 (0.02, 0.36)		0.12 (0.02, 0.21)	
*AP*			0.09 (0.01, 0.18)		0.07 (0.01, 0.13)	
*S*			1.23 (1.01, 1.50)		1.23 (1.01, 1.51)	
Cerebrovascular disease						
*Single effect for famine exposure*			1.32 (1.24, 1.41)		1.29 (1.17, 1.42)	
*Single effect for risk factor*			1.31 (1.14, 1.52)		1.02 (0.92, 1.14)	
*Combined effect*			1.70 (1.54, 1.87)		1.36 (1.24, 1.49)	
*Multiplicative interaction*			1.26 (1.21, 1.32)		1.33 (1.23, 1.43)	
Additive interaction						
*RERI*			0.06 (–0.17, 0.29)		0.04 (–0.09, 0.18)	
*AP*			0.04 (–0.01, 0.17)		0.03 (–0.07, 0.13)	
*S*			1.10 (0.77, 1.57)		1.14 (0.73, 1.78)	
Diabetic retinopathy						
*Single effect for famine exposure*			1.04 (1.00, 1.08)		1.01 (0.95, 1.06)	
*Single effect for risk factor*			1.09 (0.99, 1.19)		1.32 (1.24, 1.40)	
*Combined effect*			1.24 (1.16, 1.32)		1.20 (1.14, 1.27)	
*Multiplicative interaction*			1.09 (1.060, 1.12)		1.10 (0.94, 1.15)	
Additive interaction						
*RERI*			0.11 (–0.01, 0.23)		–0.12 (–0.31, 0.07)	
*AP*			0.09 (–0.01, 0.19)		–0.10 (–0.26, 0.05)	
*S*			1.89 (0.76, 4.69)		0.63 (0.35, 1.11)	

AP – attributable proportion due to interaction, CI – confidence interval, OR – odds ratio, RESI – relative excess risk due to interaction, S – synergy index

*Adjusted for sex, systolic and diastolic blood pressure, low-density lipoprotein cholesterol, HbA1c, diabetes duration, economic status in adulthood, metformin use, and glucagon-like peptide one receptor agonist.

†Adjusted for sex, body mass index, systolic and diastolic blood pressure, low-density lipoprotein cholesterol, HbA1c, diabetes duration, metformin use, and glucagon-like peptide one receptor agonist.

## DISCUSSION

This study utilised cross-sectional data from a nationwide population of over 200 000 individuals with T2D to investigate the impact of early-life famine exposure on the risk of developing diabetic complications in adulthood. After adjusting the confounding factors, we found that individuals with early-life famine exposure had increased risks of developing coronary heart disease, cerebrovascular disease, and diabetic retinopathy compared to those without exposure. Sex, obesity, severity of early-life famine exposure, socioeconomic status in adulthood, and duration of diabetes might modify the effects of early-life famine exposure on these diseases. Further interaction analyses indicated that obesity and high economic status might further exacerbate the risk of diabetic complications associated with early-life exposure to famine

The concept of DOHaD proposes the important concept of the first 1000 days of life, which suggests that the early stages of life (the first 1000 days) are crucial for influencing the risk of non-communicable diseases in adulthood, as both the intrauterine and early postnatal environments may alter long-term health outcomes [17]. In the Dutch famine, survivors exposed to famine in early life were found to have a higher risk of obesity and elevated blood glucose levels in adulthood, with a significantly increased risk of coronary heart disease in later life [18,19]. At the same time, as a good model for studying Asian populations, China's three-year natural disasters have provided much information to reveal the relationship between early-life famine exposure and cardiovascular metabolic risks in adulthood. A study based on the general population showed that individuals exposed to famine during the foetal period had a higher risk of ischemic stroke in adulthood (hazard ratio (HR) = 1.45; 95% CI = 1.14, 1.84) [20]. Another cross-sectional study showed that early-life famine exposure further enhanced the cardiovascular risks mediated by diabetes, including coronary heart disease, angina, congestive heart failure, and cardiac arrest [21]. A meta-analysis revealed that individuals exposed to famine during the foetal and childhood periods had a significantly higher risk of developing T2D, metabolic syndrome, hypertension, dyslipidaemia, overweight or obesity, coronary heart disease, and moderate to severe non-alcoholic fatty liver disease in adulthood, compared to unexposed individuals [22].

By using the cross-sectional data of over 200 000 individuals with T2D, the increased risks of coronary heart disease and cerebrovascular disease in adulthood were observed in diabetic patients exposed to early-life famine compared to the unexposed group, which was consistent with previous conclusions in the general populations. The Netherlands famine family study indicated that individuals exposed to famine before birth had different DNA methylation levels in genes than their siblings who were not exposed to famine [23–25]. It is assumed that epigenetic modifications might be involved in metabolic reprogramming. However, the nutrition transition in later life, including a high intake of fats, added sugars, animal-based foods, and refined carbohydrates, accompanied by reduced physical activity and increased sedentary behaviour, might lead to a mismatch with the anticipated living conditions during early-life exposure to famine, which might increase cardiovascular risks in later life [25].

Limited research data are available on the impact of early-life famine exposure on microvascular complications of diabetes. We found that in individuals with T2D, early-life famine exposure was associated with an increased risk of diabetic retinopathy and a decreased risk of diabetes kidney disease in adulthood. Early-life nutritional deprivation could interfere with vascular development processes and impair endothelial cells’ self-regulation and repair functions, making them more susceptible to damage from high oxidative stress environments in later life. This could explain why individuals with T2D who experienced famine might be more likely to develop diabetic retinopathy [26]. However, we also found a decreased risk of diabetes and kidney disease in this population, which might be a phenomenon of survivor bias. However, this hypothesis requires further validation in future investigations. Additionally, information about the use of antihypertensive medications was lacking in our studies. Certain antihypertensive agents with renal benefits, such as angiotensin-converting enzyme inhibitors, might also influence the results of this study. Further analysis with appropriate adjustments is needed in future research.

The occurrence of diabetic peripheral neuropathy and diabetic foot is even more complex. The occurrence and development of diabetic peripheral neuropathy and diabetic foot largely depend on the level of blood glucose control and the duration of diabetes in the population with T2D [27,28]. Although the initial analysis showed an increasing trend in the risk of diabetic peripheral neuropathy and diabetic foot in the early-life famine exposure group, after adjusting for confounding factors such as the duration of diabetes and HbA1c levels, this study did not find a significant impact of early-life famine exposure on the risks of diabetic peripheral neuropathy and diabetic foot in adulthood.

This study also conducted a sensitivity analysis regarding the severity of famines. The analysis results showed that compared to individuals with exposure to relatively mild famines, individuals with exposure to more severe famines had a significantly increased risk of diabetic retinopathy and diabetic nephropathy in adulthood. In the China National Nutrition and Health Survey, the researchers found that compared to the unexposed group, the increased risk of hyperglycaemia and metabolic syndrome were only observed in individuals encountering severe early-life famine exposure, while such phenomena were absent in areas with less severe famine exposure [29,30]. Similarly, the China Health and Retirement Longitudinal Study study observed an increased risk of cardiovascular diseases only in regions with severe famine exposure [31]. However, unlike previous research, our study observed a significant reduction in the risk of coronary heart disease in individuals with T2D born in areas with more severe famine exposure. This might also be influenced by survivor bias. The premature death might conceal the influence of famine severity.

The interaction analysis showed that obesity might further exacerbate the risk of diabetic complications associated with early-life exposure to famine. This suggested that cardiovascular metabolic risks in adulthood were influenced by early-life nutritional status and postnatal nutritional status. Postnatal overnutrition failed to mitigate the increased metabolic risks caused by early-life nutritional deficiencies and had an additional overlapping synergistic effect on cardiovascular metabolic risks. Therefore, weight management is important in reducing the cardiovascular metabolic risks for diabetic individuals with early-life famine exposure.

Moreover, the interaction between early-life famine exposure and economic status in adulthood showed a synergistic effect on the risk of coronary heart disease in individuals with T2D. It might be associated with the higher prevalence of unhealthy lifestyles in regions with higher economic levels in China [32,33]. Since the start of China’s reform and opening up in 1978, the birth cohort of 1959–61 experienced changes in lifestyle, with western fast food and sedentary habits entering the lives of ordinary people. All these alterations further increased the metabolic risks in individuals exposed to early-life famine, which was proposed as a ‘double-hit’ theory [34]. Therefore, early-life nutrition status and acquired lifestyle are important factors influencing the cardiovascular risks associated with T2D.

This study also has some limitations. First, due to the limited availability of information, this study did not use participants’ birth dates as a proxy variable for exposure grouping. Instead, participants were directly divided based on age, which might have led to some individuals born between 1 January and 30 September 1962 being incorrectly included in the unexposed group. These individuals might have been affected to varying degrees by famine exposure during the foetal period. In the future, more precise famine exposure grouping will be needed to further validate the conclusions of this study. Also, the famine exposure was estimated based on ecological data, which could only be treated as a proxy for the famine data. The results should be interpreted with caution. Second, although the study’s independent variable grouping was based on exposure to famine in early life rather than participants' age, there was a high degree of collinearity between famine exposure grouping and participants’ age. This resulted in an age mismatch between different famine exposure groups, and the confounding effect of ageing on the risk of cardiovascular, metabolic diseases and diabetes complications might interfere with our analysis of the metabolic effects of early-life famine exposure. However, as the three years of natural disasters in China caused nationwide famines, with hardly any region escaping from the hardship, it was difficult to find a population within the same birth year who did not experience famine exposure. In order to minimise the influence of age differences on this study, the years from 1962 to 1964 were selected as the unexposed group’s year range, thus reducing the age gap between the exposure groups (approximately three years difference between adjacent groups). Additionally, this study conducted sensitivity analyses for famine exposure severity within different famine exposure groups, further controlling for the bias caused by age differences. In previous studies on Dutch famines, researchers did not find age factors to significantly impact the metabolic effects of early-life famine exposure [35]. However, in the future, well-designed cohort studies will still be needed to further correct the influence of age by comparing the cumulative incidence rates of different clinical outcomes among various famine exposure groups. Additionally, it was assumed in the analysis that participants’ birthplace remained consistent with their place of residence. Potential population migration could lead to deviations in the coding of birthplace in certain regions. To further control for the influence of population migration on the study’s conclusions, the population migration rate was defined using the proportion of birthplace to current residence in the 2010 sixth census. The national population migration rate was 8.05%, which was not high. In the stratified analysis of regions with low population migration rates, we further verified the impact of early-life famine exposure on the risk of cardiovascular and metabolic diseases and related complications in individuals with T2D. This enhanced the reliability of the study's findings. Due to the CNHSS study’s original design to understand blood glucose control among individuals with T2D, there was limited coverage of demographic and sociological information for this population. Although this study included supplementary analysis using adult economic status, future studies should collect information related to lifestyle behaviour patterns (such as smoking status, alcohol consumption, dietary patterns, lack of physical exercise, rural or urban residence, educational level, occupation, etc.) to improve the accuracy of the analysis. Additionally, future investigations should also adjust for the use of antiplatelet, antihypertensive and lipid-lowering medications among individuals with T2D. A dedicated prospective cohort study might be able to address these concerns in the future, which hopefully to reexamine and validate our findings.

## CONCLUSIONS

Early-life exposure to famine in patients with T2D was associated with increased risks of coronary heart disease, cerebrovascular disease, diabetic retinopathy and a reduced risk of diabetic kidney disease in adulthood. Obesity and high economic status might further exacerbate the risk of diabetic complications associated with early-life exposure to famine. Improving early-life nutritional status may promote better risk prevention and management of diabetic complications in patients with T2D.

## Additional material


Online Supplementary Document
