# Supplementary material for: The association between exposure to famine in early life and risks of diabetic complications in adult patients with type two diabetes
Source: J Glob Health. 2024 Sep 20;14:04167. doi: 10.7189/jogh.14.04167 (PMC11414423; doi:10.7189/jogh.14.04167)

Supplementary Material

Supplementary Table

Table S1. Mortality for each province/municipality/autonomous region during 1956-1962 (per 1000 person)
Table S2. Economic status for each province/municipality/autonomous region during 2019-2013
Table S3. Migration rate for each province/municipality/autonomous region
Table S4. The definitions of diabetic complications in CNHSS
Table S5. Clinical characteristics of different famine-exposed subgroups

Supplementary Figure

Figure S1. The sensitivity analyses for the association between early-life famine exposure and diabetic complications in the adulthood
Figure S2. The sensitivity analyses for the association between early-life famine exposure and diabetic complications in the adulthood by recruiting year

Table S1. Mortality for each province/municipality/autonomous region during 1956-1962 (per 1000 person)

Year	1956	1957	1958	1959	1960	1961	1962	Average mortality	Relative change in mortality
China	11.4	10.8	12	14.6	25.4	14.2	10	11.4	**122.8**
Tianjin	8.8	9.4	8.7	9.9	10.3	7.4	9	14.9	14.9
Shanxi	11.6	12.7	11.7	12.8	14.2	12.2	11.3	12	18.3
Shaanxi	9.9	10.3	11	12.7	12.3	8.7	9.4	10.4	22.1
Shanghai	6.8	6	5.9	6.9	6.8	7.7	7.3	6.2	23.5
Inner Mongolia	7.9	10.5	7.9	11	9.5	8.8	9	8.8	25.5
Zhejiang	9.5	9.3	9.2	10.8	11.9	9.8	8.6	9.3	27.5
Heilongjiang	10.1	10.5	9.2	12.8	10.5	11.1	8.6	9.9	28.9
Ningxia	10.6	11.1	15	15.8	13.9	10.7	8.5	12.2	29.2
Beijing	7.7	8.2	8.1	9.6	9.2	10.8	8.7	8	35
Xinjiang	13.9	13.9	13.9	18.8	NA	NA	9.7	13.9	35.3
Jiangxi	12.5	11.5	11.3	13	16.1	11.5	11	11.8	36.8
Hebei	11.3	11.3	10.3	12.3	15.8	13.6	9.1	11	44.1
Yunnan	15.2	16.3	21.6	18	26.3	11.9	10.9	17.7	48.6
Jilin	7.5	9.1	9.1	13.4	10.1	12.1	10	8.6	56.4
Guangdong	11.2	8.4	9.1	11.7	15.1	10.7	9.3	9.6	57.8
Jiangsu	13	10.3	9.4	14.6	18.4	13.4	10.4	10.9	68.8
Shandong	12.1	12.1	12.8	18.2	23.6	18.5	12.4	12.3	91.4
Fujian	8.4	7.9	7.5	7.9	15.3	11.9	8.3	7.9	92.9
Hubei	10.9	9.6	9.6	14.5	21.2	9.2	8.8	10	**111.3**

Liaoning	6.6	9.4	8.8	11.8	11.5	17.5	8.5	8.3	**111.7**
Guangxi	12.5	12.4	11.7	17.5	29.5	19.5	10.3	12.2	**141.8**
Hunan	11.5	10.4	11.7	13	29.4	17.5	10.4	11.2	**162.5**
Gansu	10.8	11.3	21.1	17.4	41.3	11.5	8.2	14.4	**186.8**
Henan	14	11.8	12.7	14.1	39.6	10.2	8	12.8	**208.6**
Sichuan	10.4	12.1	25.2	47	54	29.4	14.6	15.9	**239.6**
Qinghai	9.4	10.4	13	16.6	40.7	11.7	5.4	10.9	**272.3**
Guizhou	13	12.4	15.3	20.3	52.3	23.3	11.6	13.6	**285.5**
Anhui	14.3	9.1	12.4	16.7	68.6	8.1	8.2	11.9	**474.9**

NA, not available.

Table S2. Economic status for each province/municipality/autonomous region during 2019-2013

Year	High economic status	Low economic status
2009	Shanghai, Beijing, Tianjin, Zhejiang, Guangdong, Jiangsu, Fujian, Liaoning, Shandong	Inner Mongolia, Chongqing, Jilin, Hebei, Hubei, Hunan, Heilongjiang, Hainan, Jiangxi, Shanxi, Ningxia, Anhui, Guangxi, Henan, Sichuan, Shaanxi, Qinghai, Xinjiang, Yunnan, Gansu, Guizhou, Xizang
2010	Shanghai, Beijing, Tianjin, Zhejiang, Guangdong, Jiangsu, Fujian, Liaoning, Shandong	Inner Mongolia, Chongqing, Jilin, Hebei, Hubei, Hunan, Heilongjiang, Hainan, Jiangxi, Shanxi, Ningxia, Anhui, Guangxi, Henan, Sichuan, Shaanxi, Qinghai, Xinjiang, Yunnan, Gansu, Guizhou, Xizang
2011	Shanghai, Beijing, Tianjin, Zhejiang, Guangdong, Jiangsu, Fujian, Liaoning, Shandong	Inner Mongolia, Chongqing, Jilin, Hebei, Hubei, Hunan, Heilongjiang, Hainan, Jiangxi, Shanxi, Ningxia, Anhui, Guangxi, Henan, Sichuan, Shaanxi, Qinghai, Xinjiang, Yunnan, Gansu, Guizhou, Xizang
2012	Shanghai, Beijing, Tianjin, Zhejiang, Guangdong, Jiangsu, Fujian, Liaoning, Shandong, Inner Mongolia	Chongqing, Jilin, Hebei, Hubei, Hunan, Heilongjiang, Hainan, Jiangxi, Shanxi, Ningxia, Anhui, Guangxi, Henan, Sichuan, Shaanxi, Qinghai, Xinjiang, Yunnan, Gansu, Guizhou, Xizang
2013	Shanghai, Beijing, Tianjin, Zhejiang, Guangdong, Jiangsu, Fujian, Liaoning, Shandong, Inner Mongolia	Chongqing, Jilin, Hebei, Hubei, Hunan, Heilongjiang, Hainan, Jiangxi, Shanxi, Ningxia, Anhui, Guangxi, Henan, Sichuan, Shaanxi, Qinghai, Xinjiang, Yunnan, Gansu, Guizhou, Xizang

Table S3. Migration rate for each province/municipality/autonomous region

Region	Birthplace same as current residence	Birthplace different from current residence	Total population	Migration rate (%)
China	127339585	117094400	10245185	8.05
Beijing	1849475	1010144	839331	**45.4**
Tianjin	1127589	863304	264285	**23.4**
Hebei	7037620	6825067	212553	3.02
Shanxi	3477805	3368785	109020	3.13
Inner Mongolia	2310941	2098083	212858	9.21
Liaoning	4252076	3981205	270871	6.37
Jilin	2551123	2455198	95925	3.76
Heilongjiang	3465051	3270560	194491	5.61
Shanghai	2253525	1243754	1009771	**44.81**
Jiangsu	7577122	6785998	791124	**10.44**
Zhejiang	5400348	4124252	1276096	**23.63**
Anhui	5312628	5183564	129064	2.43
Fujian	3477491	3061703	415788	**11.96**
Jiangxi	4251692	4149719	101973	2.40
Shandong	9272503	9021656	250847	2.71
Henan	9224288	9114197	110091	1.19
Hubei	5226904	5049835	177069	3.39
Hunan	6096586	6005456	91130	1.49
Guangdong	9676589	7445759	2230830	**23.05**
Guangxi	4362551	4260689	101862	2.33
Hainan	826560	752593	73967	8.95

Chongqing	2609882	2482494	127388	4.88
Sichuan	8161604	7987753	173851	2.13
Guizhou	3332265	3213388	118877	3.57
Yunnan	4467537	4331413	136124	3.05
Xizang	265904	246513	19391	7.29
Shaanxi	3614887	3456731	158156	4.38
Gansu	2623094	2544097	78997	3.01
Qinghai	535412	484577	50835	9.49
Ningxia	611957	550493	61464	**10.04**
Xinjiang	2086576	1725420	361156	**17.31**

Table S4. The definitions of diabetic complications in CNHSS

Diabetic complications	Definitions
Coronary heart disease	Based on retrospective medical records, these cases include individuals who developed any of the following after a diagnosis of type 2 diabetes: ischemic heart disease with abnormal electrocardiogram or positive exercise stress test, myocardial infarction with dynamic changes in electrocardiogram or enzymatic alterations, post-coronary artery bypass grafting, post-percutaneous coronary intervention, or post-endarterectomy for atherosclerotic plaques.
Cerebrovascular disease	Based on retrospective medical records, these cases include individuals who developed ischemic stroke, subarachnoid hemorrhage, or intracranial hemorrhage after a diagnosis of type 2 diabetes.
Diabetic kidney disease	Clinical diagnosis is usually made based on increased urinary albumin excretion rate (UAER) and/or decreased estimated glomerular filtration rate (eGFR), while excluding other causes of chronic kidney disease. An UAER ≥20 μg/min or UAER ≥30 mg/24 h, along with exclusion of other causes of kidney damage, urinary tract infections, and hematuria, indicates increased urinary albumin excretion. An eGFR <60 ml/min/1.73 m^2 indicates decreased glomerular filtration rate.
Diabetic peripheral neuropathy	This refers to the occurrence of neuropathy at the time of diabetes diagnosis or afterward, with clinical symptoms and signs consistent with diabetic peripheral neuropathy (such as symmetrical numbness, burning sensation, etc.). At least two abnormal findings from the following five tests are required for diagnosis: abnormal temperature sensation, nylon monofilament examination indicating decreased or absent foot sensation, abnormal vibration sensation, absent ankle reflex, or abnormalities in nerve conduction velocity.
Diabetic foot	This refers to foot ulcers, including both healed and non-healed ulcers, as well as amputations resulting from ulcer deterioration

Table S5. Clinical characteristics of different famine-exposed subgroups

Parameter	Unexposed (n=72723)	Fetal exposed (n=70852)	Infant exposed (n=93616)	Early-life exposed (n=164468)	P[a]
Age (years old)	48.36±1.51[bc]	51.35±1.57[cd]	54.45±1.56[bd]	53.11±2.20	<0.001
Male percentage (%)	56.3[bc]	52.7[cd]	51.0[bd]	51.8	<0.001
Height (cm)	167.07±7.76[bc]	166.31±7.78[cd]	165.94±7.77[bd]	166.10±7.77	<0.001
Body weight (kg)	68.30±10.93[bc]	67.37±10.55[cd]	67.08±10.39[bd]	67.21±10.46	<0.001
BMI (kg/m^2)	24.41±3.16[bc]	24.30±3.08[d]	24.31±3.10[d]	24.31±3.10	<0.001
SBP (mmHg)	129.37±14.13[bc]	130.05±14.21[cd]	130.63±14.45[bd]	130.38±14.35	<0.001
DBP (mmHg)	81.33±10.32[bc]	81.62±10.52[d]	81.70±10.79[d]	81.66±10.67	<0.001
LDL-C (mmol/L)	2.98±4.89	3.00±5.13	2.99±4.99	3.00±6.62	0.793
FBG (mmol/L)	8.05±2.39[bc]	8.00±2.32[cd]	7.97±2.33[bd]	7.98±2.32	<0.001
2h-PBG (mmol/L)	11.25±3.69[c]	11.25±3.60[c]	11.19±3.59[bd]	11.21±3.59	<0.001
HbA1c (%)	7.82±1.67[bc]	7.87±1.71[d]	7.86±1.69[d]	7.86±1.70	<0.001
Targeted HbA1c control rate (%)	34.1[b]	33.0[cd]	34.1[b]	33.7	0.057
Targeted BP control rate (%)	73.8[bc]	72.3[d]	71.8[d]	72.0	<0.001
Targeted LDL-C control rate (%)	42.7	42.9c	42.3[b]	42.5	0.550
Diabetes duration (years)	3.98±3.76[bc]	4.32±3.95[cd]	4.73±8.27[bd]	4.55±7.74	<0.001

BMI, body mass index; SBP, systolic blood pressure; DBP, diastolic blood pressure; LDL-C, low density lipoprotein cholesterol; FBG, fasting blood glucose; 2h-PBG, 2-hour prandial blood glucose; HbA1c, glycated hemoglobin. Targeted HbA1c control was defined as HbA1c <7%; targeted BP control was defined as BP <130/80mmHg; targeted LDL-C control was defined as LDL-C <2.6mmol/L. a, the comparison between the early-life exposed subgroup and unexposed subgroup; b, compared with fetal exposed subgroup, adjusted P<0.05; c, compared with infant exposed subgroup, adjusted P<0.05; d, compared with unexposed subgroup, adjusted P<0.05. All P value were adjusted for multiple comparisons.

Figure S1. The sensitivity analyses for the association between early-life famine exposure and diabetic complications in the adulthood

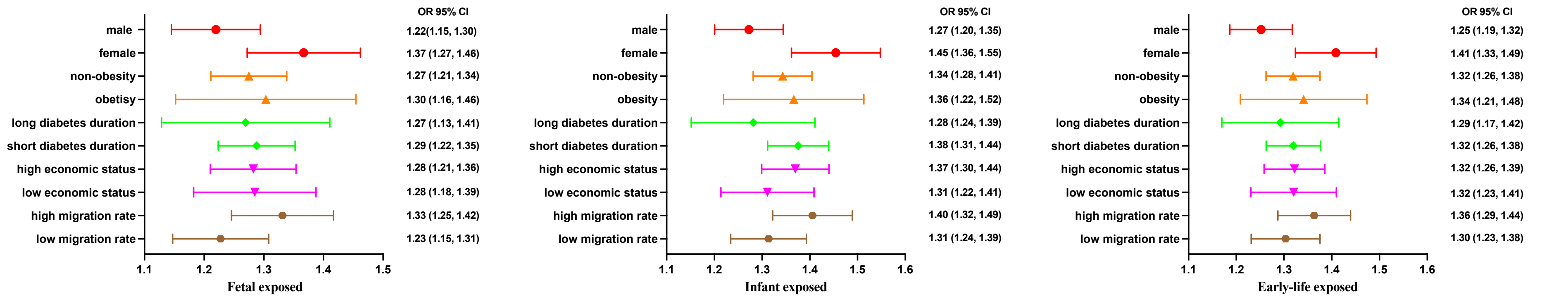

(A) coronary heart disease

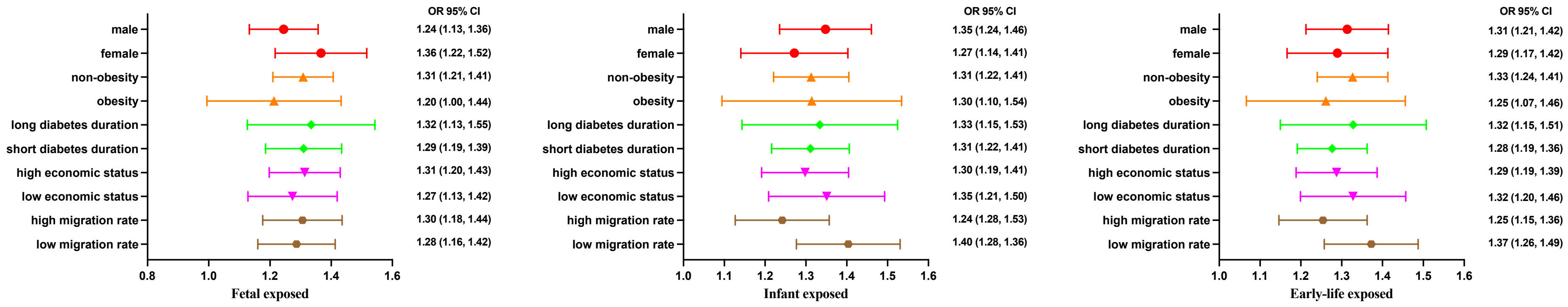

(B) cerebrovascular disease

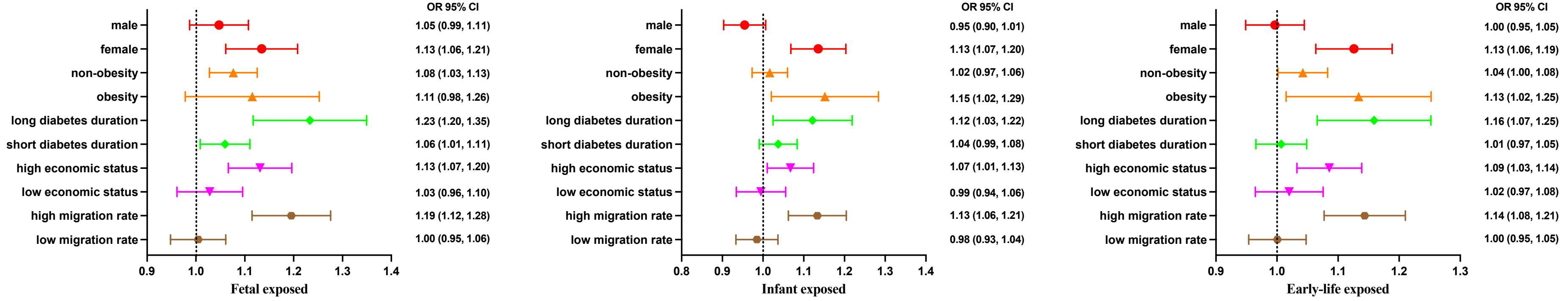

(C) diabetic retinopathy

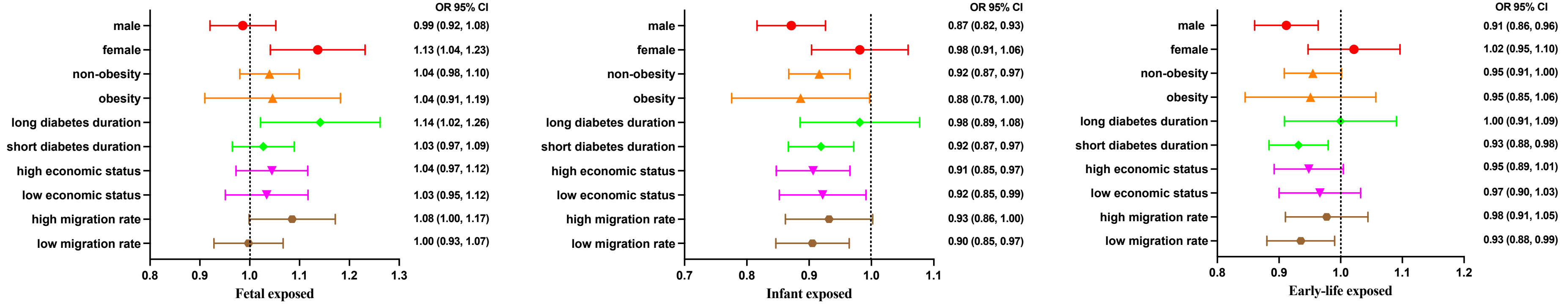

(D) diabetic kidney disease

Figure S2. The sensitivity analyses for the association between early-life famine exposure and diabetic complications in the adulthood by recruiting year

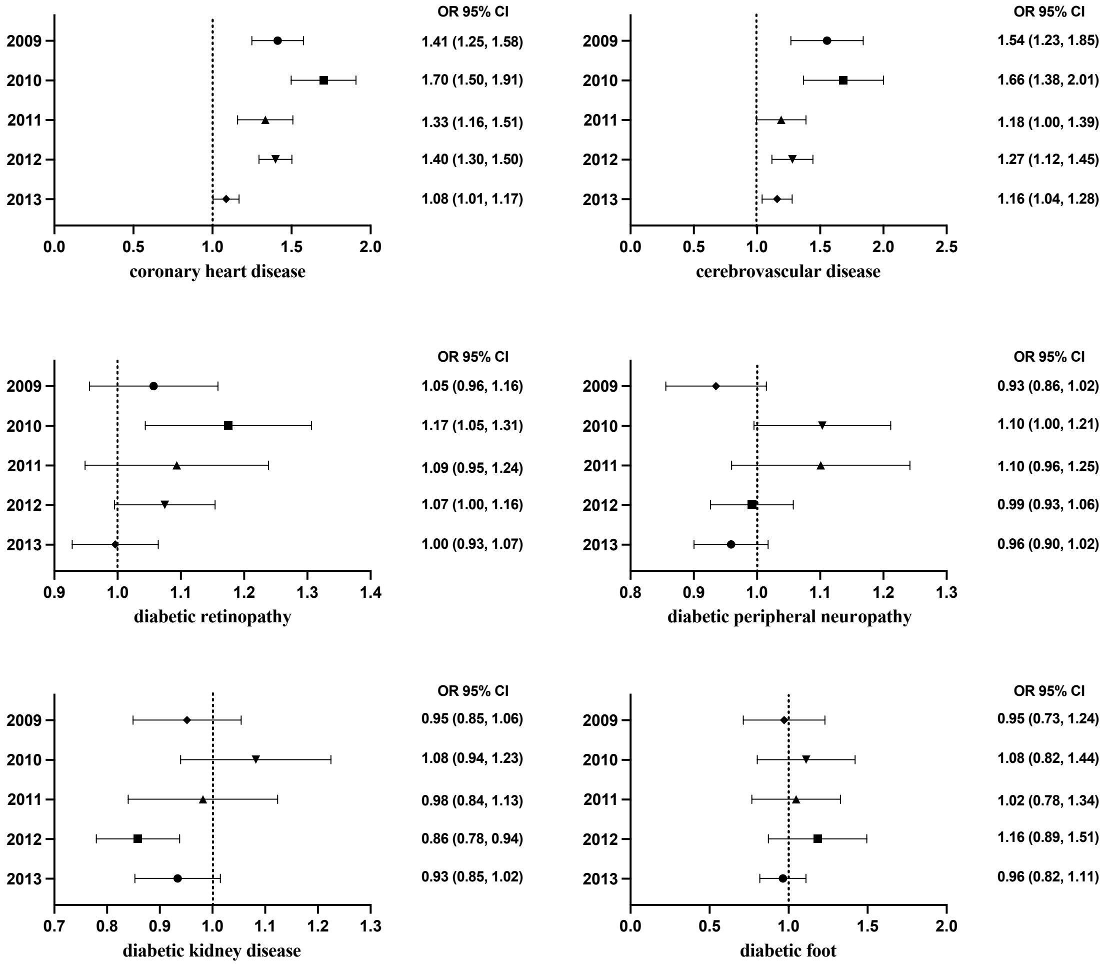

Supplement: Online Supplementary Document [file jogh-14-04167-s001.pdf]
